# Supplementary figures and images for: Screening conditions and constructs for attempted genetic transformation of C. elegans by Agrobacterium
Source: PLoS One. 2025 May 27;20(5):e0325060. doi: 10.1371/journal.pone.0325060 (PMC12111652; doi:10.1371/journal.pone.0325060)

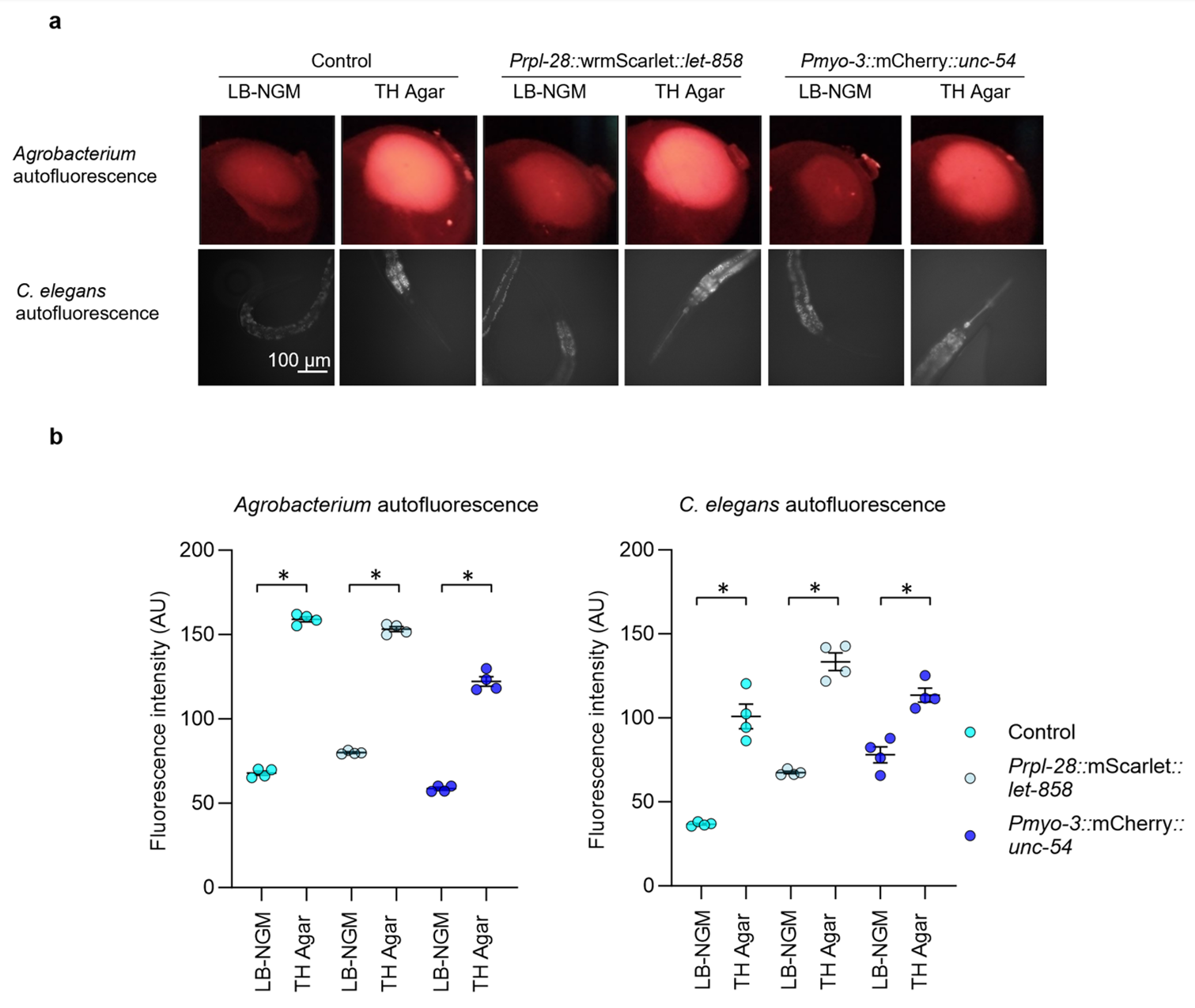

Supplement: S2 Fig — (a) Autofluorescence of Agrobacterium containing different T-binary vectors grown on LB-NGM or Todd Hewitt (TH) agar was assessed by pelleting a known number of cells and visualizing the pellet in an Eppendorf tube using widefield fluorescence microscopy. Autofluorescence of C. elegans fed Agrobacterium grown on LB-NGM or TH agar was visualized using widefield fluorescence microscopy, using the DsRed channel. (b) Quantification of autofluorescence from Agrobacterium and C. elegans grown on LB-NGM or TH agar (Mann–Whitney test, n = 4, error bars represent mean + /- SEM). (TIF) [file pone.0325060.s002.tif]

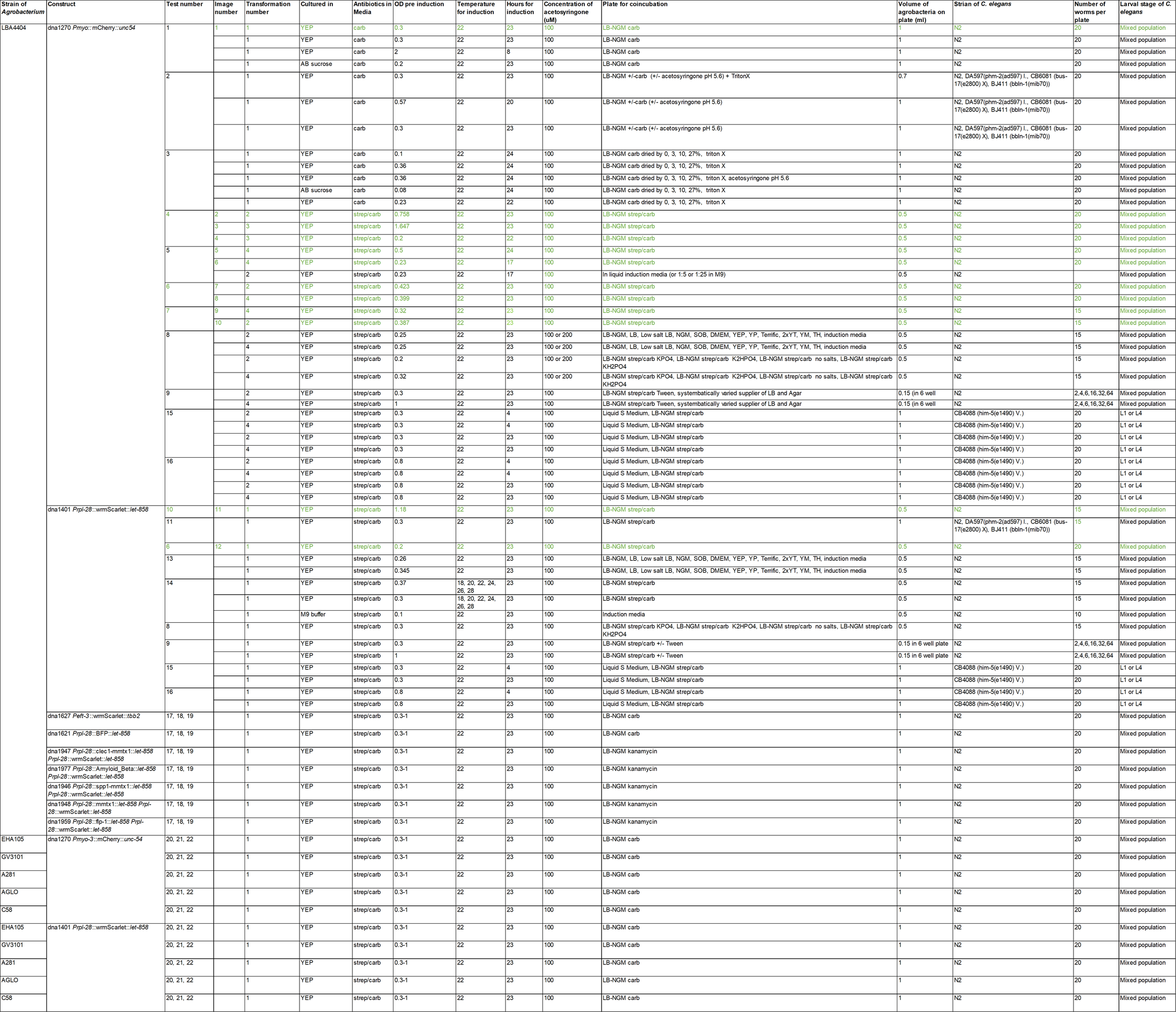

Supplement: S1 Table — C. elegans fed Agrobacterium carrying T-binary vectors engineered for fluorescence expression in worms occasionally exhibited fluorescence in specific body regions, which appeared noticeably brighter than the typically observed autofluorescence. Experimental conditions associated with these phenotypes are highlighted in green, and representative images are displayed in S3 Fig. (TIF) [file pone.0325060.s003.tif]

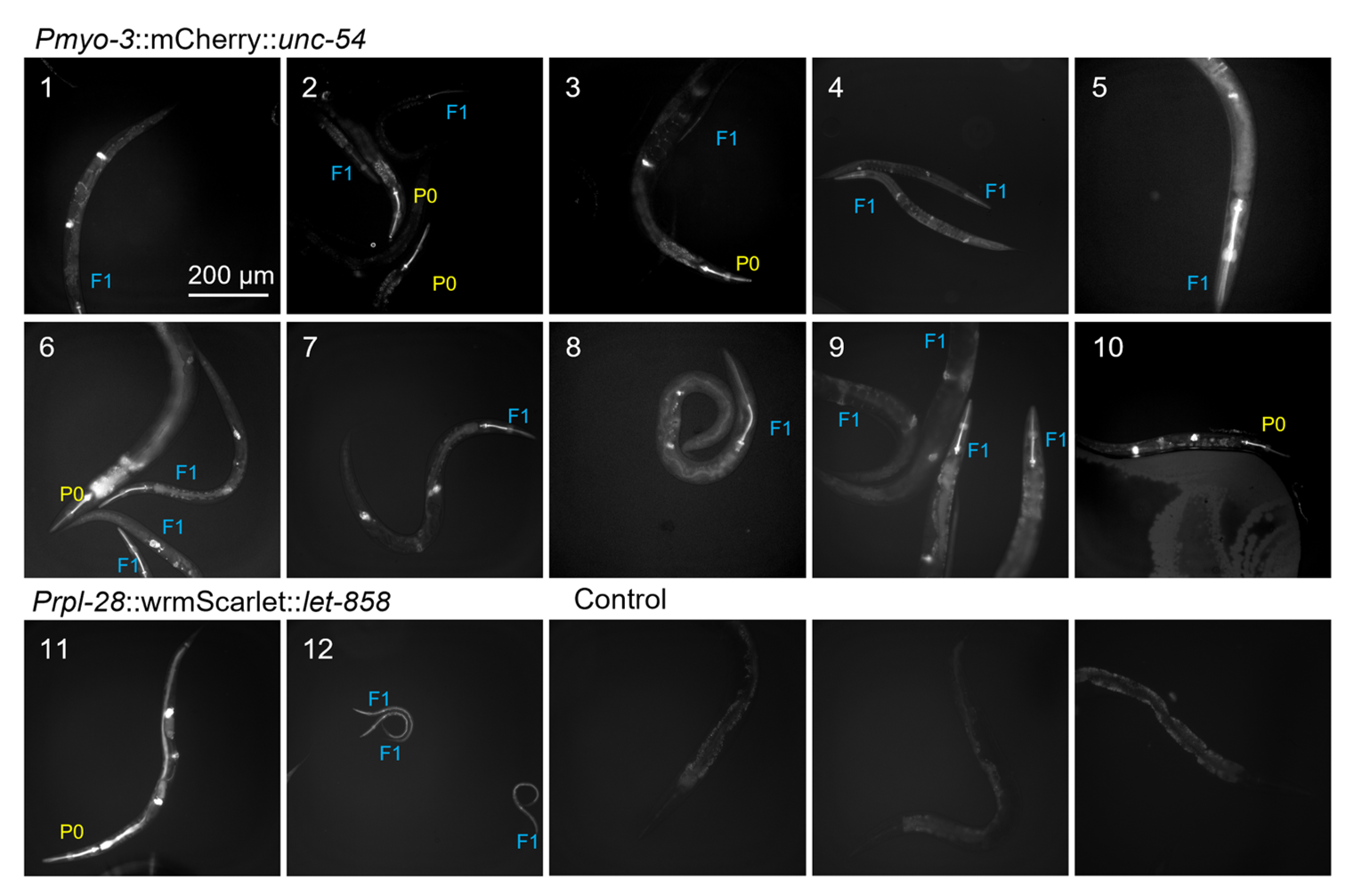

Supplement: S3 Fig — Images were captured using widefield fluorescence microscopy in the DsRed channel. Wild-type worms without Agrobacterium treatment served as controls, with three representative images shown. (TIF) [file pone.0325060.s004.tif]

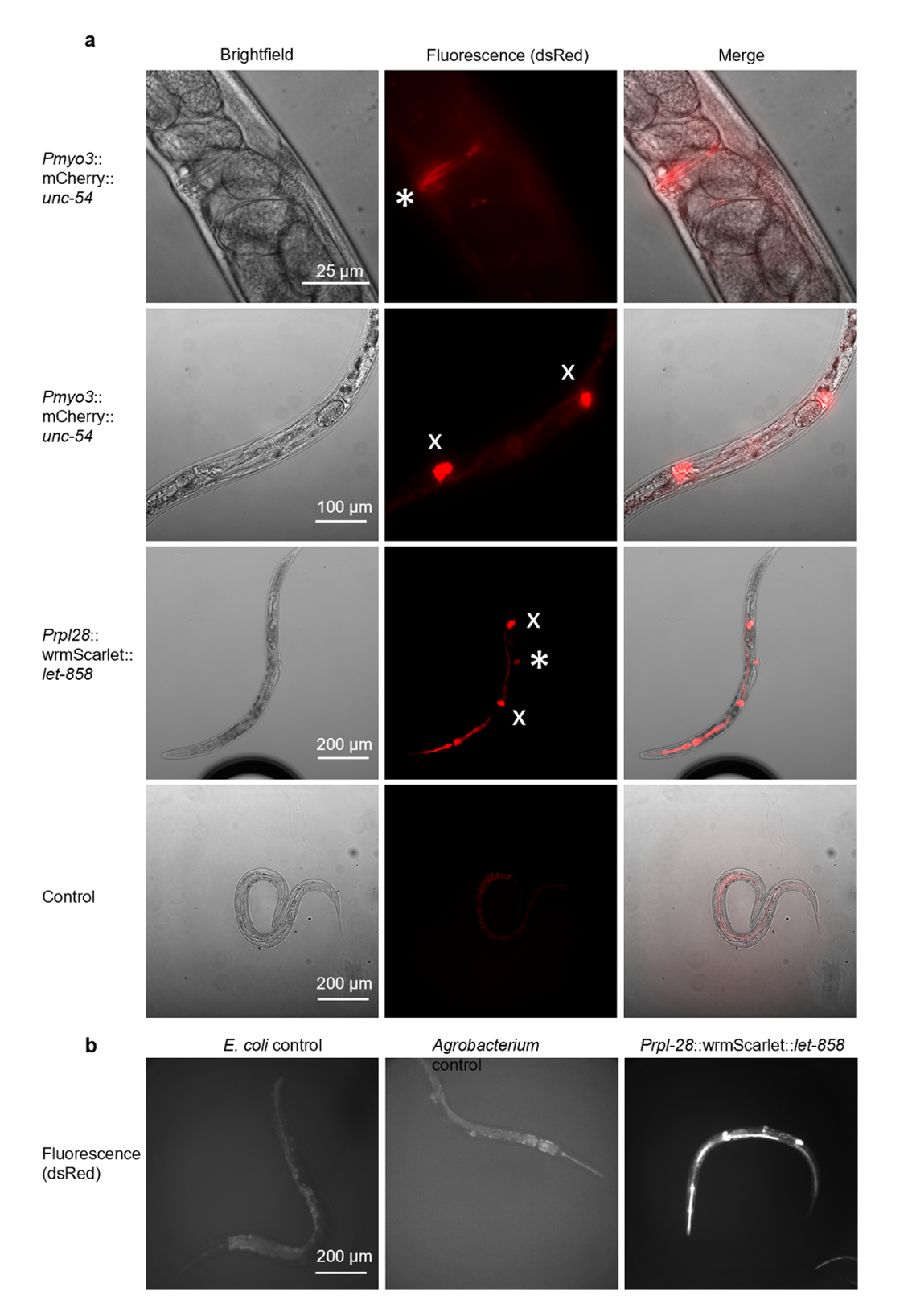

Supplement: S4 Fig — C. elegans fed Agrobacterium carrying T-DNA engineered for fluorescence expression occasionally exhibited atypically bright fluorescence in the vulva and in two regions approximately 100 µm anterior and posterior to the vulva. (a) Fluorescence was detected in the vulva (labeled with *), as well as in two lateral regions (labeled with x), following exposure to Agrobacterium strain LBA4404 containing either the Pmyo-3::mCherry::unc-54 or Prpl-28::wrmscarlet::let-858 T-binary vector. Control worms exposed to Agrobacterium lacking the T-binary vector are also shown. (b) Autofluorescence observed in C. elegans fed E. coli (OP50) or Agrobacterium lacking the T-binary vector is compared to the fluorescence observed in C. elegans fed Agrobacterium carrying the Prpl-28::wrmscarlet::let-858 construct. Images were captured using widefield fluorescence microscopy in the DsRed channel and brightfield microscopy. (TIF) [file pone.0325060.s005.tif]

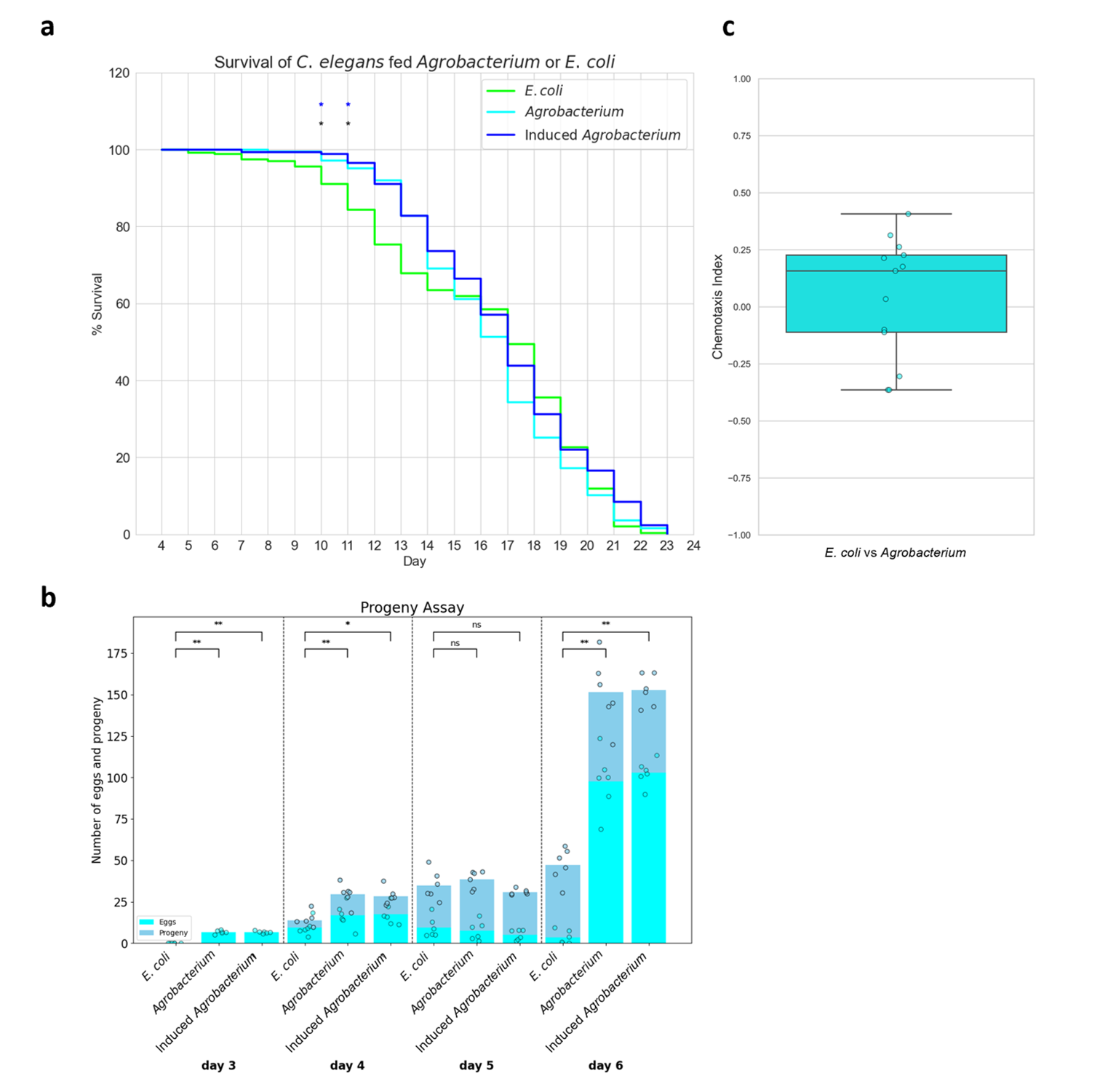

Supplement: S5 Fig — (a) Survival curves of C. elegans fed E. coli (OP50), uninduced Agrobacterium, or induced Agrobacterium (Kruskal–Wallis test with Dunn’s post hoc test, n = 6). (b) Quantification of eggs and progeny laid over time by C. elegans fed E. coli (OP50), induced Agrobacterium or uninduced Agrobacterium (Kruskal–Wallis test with Dunn’s post hoc test, n = 6). (c) Chemotaxis index of C. elegans comparing E. coli (OP50) against induced Agrobacterium (Mann–Whitney t test, n = 6. Box represents IQR, line shows median value). (TIF) [file pone.0325060.s006.tif]
