# Supplementary material for: Screening conditions and constructs for attempted genetic transformation of C. elegans by Agrobacterium
Source: PLoS One. 2025 May 27;20(5):e0325060. doi: 10.1371/journal.pone.0325060 (PMC12111652; doi:10.1371/journal.pone.0325060)
Supplement: Attachment — Submitted filename: Reviewers comments PONE-D-24-38923.pdf [file pone.0325060.s008.pdf]

In this manuscript (*PONE-D-24-38923*), Warren et al. present data from their attempt to establish a platform for the genetic transformation of *C. elegans* using *Agrobacterium*. This bacterial strain is a natural plant pathogen, primarily used for genetic transformation of plants, with limited studies demonstrating its use for transformation in other kingdoms and even human cell lines. The authors argue that conventional methods for genetic transformation of *C. elegans* (microinjection and biolistic bombardment) lack the potential for high-throughput and timely generation of *C. elegans* mutant libraries. To address this limitation, the authors propose using different *Agrobacterium* strains, alongside specific constructs and screening conditions, as a food source for worms to develop a simpler and more efficient high-throughput transgenesis protocol. However, as the authors acknowledge, their attempts were unsuccessful. Nonetheless, they present their data to support the scientific community by providing constructs, conditions, and optimization suggestions, aiming to serve as a foundation for future successful endeavors.

Also, using established systems for phenotyping *C. elegans* behavior, the authors tested different constructs with genes responsible for inducing paralysis. Despite these constructs failing to induce paralysis due to unsuccessful transformation from *Agrobacterium*, the authors assessed worm behavior in terms of forward and paused motion frequencies.

Overall, the manuscript introduces a novel yet unsuccessful approach for gene transfer from *Agrobacterium* to *C. elegans*. Given the significance of *C. elegans* as a model organism in genetic studies, a contribution acknowledged by four Nobel Prizes, developing a simple and high-throughput method for genetic manipulation is of immense interest. While the authors provide an honest representation of their data, some claims require justification to enhance the reliability of the study and ensure its suitability for publication. Below, I provided some suggestions to improve the manuscript's rigor and address gaps in study design and presentation.

Major Points:

1. The authors state in the Results section: "In certain conditions, worms fed *Agrobacterium* containing T-DNA designed to introduce wrmScarlet expression did display **remarkable fluorescence** (Fig. 3c)." Additionally, they claim: "Bright fluorescence appeared to localize to the pharynx, the vulva, and two regions on the ventral side of the nematode approximately 100 µm anterior and posterior to the vulva, consistent with the spermatheca." Similar claims appear in the Discussion, suggesting that *Agrobacterium* has the potential to target the germline of *C. elegans* for generating heritable transformants: "Despite our lack of success, we have observed Agrobacterium triggered fluorescence in the reproductive tissues of the vulva and spermatheca, suggesting that Agrobacterium has the potential to target the germline of C. elegans for the generation of heritable transformants." and "This indicates that Agrobacterium are interacting at these locations, to trigger fluorescence, rather than accumulating directly. This finding suggests that Agrobacterium-mediated transformation is a promising approach since Agrobacterium

are able to access reproductive tissues suggesting a propensity to target the germline of C. elegans for the generation of heritable transformants."

Although the authors mentioned that the data presented was not always reproducible, the data presented in Fig. 3c do not convincingly support these claims. The fluorescence attributed to the spermatheca is based on rough distance calculations. Additionally, the control worm in Fig. 3a shows autofluorescence comparable to the foci observed in Fig. 3c.

- To substantiate this claim, the authors should:
    - Provide additional evidence, such as staining with DAPI to detect sperm cell nuclei or using differential interference contrast (DIC) optics to superimpose fluorescent channels on reproductive tissues. These methods would help verify whether the fluorescence observed corresponds specifically to the spermatheca and support the claim of targeted interaction by *Agrobacterium*.
    - Reevaluate the conclusion that the observed fluorescence is a direct effect of GFP-expressing bacteria. Without stronger evidence, the assertion that *Agrobacterium* has potential for germline transformation is misleading.

2. The authors state that "*C. elegans* were able to survive and reproduce on *A. tumefaciens*, as demonstrated by the presence of progeny after 4 days." However, no supporting data are provided. The ability to produce viable progeny is crucial for successful genetic transformation.

- It is recommended that the authors conduct progeny assays to assess the number and viability of offspring when worms are fed on *A. tumefaciens* compared to *E. coli*. Include this data as supplemental material to strengthen the study.

3. While the authors' attempts at gene transfer were unsuccessful, including data on the general transformation efficiency of *A. tumefaciens* would help assess the feasibility of this approach and provide a benchmark for evaluating its potential in *C. elegans*. Including this information as supplemental data could help the scientific community better evaluate the feasibility of the proposed approach.

4. Table 1 lists "Conditions Screened for *Agrobacterium* Induction and Co-incubation with *C. elegans*." While potentially useful, the table lacks important details, likely due to insufficient study design.

- A more comprehensive table, including key experimental parameters and results, would improve the manuscript's utility for other researchers.

Minor Points:

1. Reference 14, a master's thesis analyzing host-pathogen interactions between *Agrobacterium* and *C. elegans*, does not include experiments on genetic transformation. However, this reference (although not peer-reviewed), suggests that *C. elegans* can survive on *A. tumefaciens* as a sole food source, as shown by longevity assays.

   - The current manuscript evaluates worm crawling speed and length when grown on *A. tumefaciens*. Since the cited reference includes viability assays, adding comparative data on worms grown on *E. coli* would provide better context for these observations. Additionally, examining germline morphology, cell migration, or other anatomical or physiological factors could offer deeper insights into the survival and adaptation of *C. elegans*when fed exclusively on *A. tumefaciens*.
