# Supplementary material for: Screening conditions and constructs for attempted genetic transformation of C. elegans by Agrobacterium
Source: PLoS One. 2025 May 27;20(5):e0325060. doi: 10.1371/journal.pone.0325060 (PMC12111652; doi:10.1371/journal.pone.0325060)
Supplement: Attachment — Submitted filename: Response to Reviewers.docx [file pone.0325060.s010.docx]

Dear Dr Srinivasan

Thank you for the opportunity to revise and improve our manuscript in accordance with PLOS ONE's requirements. We sincerely appreciate the thoughtful feedback provided by the reviewers and have carefully addressed each of their comments. In this rebuttal letter, we document the specific changes made in response to the four reviewers’ suggestions, for clarity our responses are in blue, and changes to the manuscript are also included in the document “Revised Manuscript with Track Changes.docx”. Some additional formatting corrections (typos, and italics etc.) have been made in the final document “Manuscript.docx”. Additionally I have uploaded and published detailed versions of the protocols on protocols.io and have included the DOI in the methods section as recommended. We hope that our responses and the revised manuscript meet the expectations of the editorial team and reviewers.

Yours Sincerely,
Eleanor Warren

Reviewer #1: This article explores the development of a novel technique for C.elegans transgene. Currently, the most common approach involves injecting DNA, protein, or RNA into the gonads of worm via microinjection to establish a transgenic line for genetic analysis. However, microinjection presents a high technical barrier and low efficiency. The authors propose an alternative approach using Agrobacterium to generate transgene, which is an intriguing idea. However, while the authors have conducted various attempts, the results indicate that transgene has not been successfully achieved. It remains unclear whether the method is inherently unfeasible or if further optimization of the experimental conditions could yield success. Key challenges include:
 1. Uncertainty regarding whether the virulence protein in Agrobacterium is effectively expressed.
 2. The host-specific nature of the Type IV secretion system (T4SS), raising doubts about whether Agrobacterium can deliver DNA and protein into worm cells via this pathway.
 3. Ambiguity around whether the virulence protein can insert T-DNA into the worm genome.
 After carefully reviewing the methods section, I suggest the following improvements:

1. Induce the expression of key virulence proteins in Agrobacterium by using the worm liquid culture method (refer to WormBook). Resuspend Agrobacterium in S-medium and include appropriate inducers to promote virulence gene expression.

Thank you for this suggestion.
We followed your advice, and attempted to induce the expression of the virulence genes by resuspending Agrobacterium in S-medium and inducing with Acetosyringone.
Strains of Agrobacterium containing T-binary vectors (pmyo-3::mCherry::Unc-54, and pRPL-28::wrmscarlet::Let-858) that have previously resulted in fluorescent *C. elegans* were tested (as well as a control strain with no T binary vector). Unfortunately no fluorescence was observed in any of these conditions.
Representative images are shown here.

Control	pmyo-3::mCherry::Unc-54	pRPL-28::wrmscarlet::Let-858

Here is a detailed version of the method I tested.
Agrobacterium was streaked to Yeast Extract Peptone agar plates containing appropriate antibiotics, and incubated for 3 days at 28°C. A single colony of Agrobacterium was used to inoculate 20 ml YEP media (with appropriate antibiotics) which was incubated overnight. Overnight cultures were diluted 1:100 in 20 ml YEP media (with appropriate antibiotics), and these cultures were incubated at 28°C and 250-rpm until the Agrobacterium reached an OD600 of 0.3 or 0.8. Agrobacterium cells were pelleted by centrifugation (5 min 4000g), before resuspension in 40 ml S-medium containing 100 µM acetosyringone, and shaking at 50-rpm at room temperature for 4 or 23 hours. 2 ml of this suspension was transferred to a well of a 24 well plate, and 20 L4 *C. elegans* (washed 3 times in M9 to remove E. coli) were added. These cultures were incubated overnight at 20°C with shaking at 200 rpm. *C. elegans* were then transferred to NGM plates seeded with OP50. Plates were incubated at 20 degrees for 4 days. Fluorescence of the C. elegans P0 generation and the progeny was observed directly on plates using an Olympus U-RFL-T microscope and on microscope slides using a Olympus IX70 fluorescence microscope.

2. Detect the expression of virulence genes using qPCR after inducing.

We appreciate the reviewer’s suggestion to detect virulence gene expression using qPCR. To design qPCR primers specific to Ti plasmid pAL4404, we attempted to extract and sequence the plasmid extracted from *Agrobacterium tumefaciens* strain LBA4404, since the sequence of pAL4404 is not publicly available. However, our sequencing efforts did not recover sufficient plasmid reads, likely due to low plasmid yield. Given the absence of a complete sequence for pAL4404, we are unfortunately currently unable to design specific primers for qPCR.

3. Use synchronized worms for transgene. Transfer L4-stage or young adult (YA) worms to induced-Agrobacterium culture from step 1, followed by overnight liquid culture. Subsequently, transfer the worms to NGM plates contain OP50.

Please see the response to point 1

4. Examine the localization of Agrobacterium in the parental P0 generation.

Thanks for your suggestion. I monitored the P0 generation for the position of GFP expressing agrobacterium. This shows the same localisation as shown in Figure 2, with *Agrobacterium* identified within the lumen of the pharynx and within the intestine of the worm. Hence I edited the manuscript to state that this localisation was observed in both the P0 generation and the progeny.

“We fed *C. elegans* *Agrobacterium* expressing GFP and observed fluorescence within the lumen of the pharynx and within the intestine of the worm both in the P0 generation and in the progeny (Fig. 2c).”

Here are images I obtained from the P0 generation

GFP	Brightfield	Merge

5. Screen for transgenic worms in the F1 progeny. Use fluorescent labeling to detect the presence of the transgene in subsequent generations.

Thank you for your suggestion. Indeed our protocol already involves screening the F1 progeny for fluorescence. I have updated the text to make sure that this is clear.

*“C. elegans* from different life stages (from L1 to adult) were exposed to induced *Agrobacterium*, and fluorescence was monitored both in the parental generation (P0) and in the progeny (F1). “

Reviewer #2: Here the authors present largely negative data, as well as resources and suggestions for further research, which could contribute to the application of Agrobacterium-mediated transformation in C. elegans. Publication of negative results is an important contribution to the scientific process, preventing others from wasting time and resources by repeating the same experiments, as well as guiding future research.

 I have no major concerns, but do have some minor comments to help improve the manuscript:
 1. There are still quite a few typos and style errors that need to be fixed prior to publication.

Thank you, I have corrected the style errors in line with the PLOS ONE requirements.

2. Statistics are missing for the data presented in Fig. 2.

Thank you for bringing this to my attention. I have added more detail to the text regarding the statistics, I have also added this information to the figure legend for Fig. 2, and I have added a section on statistics in the methods section.

“Adult *C. elegans* fed on *A. tumefaciens* were shorter in length (Fig. 2a, p<0.0001, Unpaired Mann- Whitney test), while speed was not significantly different (Fig. 2b) (Unpaired Mann- Whitney test).”

Fig. 2. Appearance of C. elegans fed on Agrobacterium. Adult C. elegans fed on Agrobacterium or E. coli were monitored for (a) length (Mann–Whitney test, n >72). (b) and speed (Mann–Whitney test, n >72. Error bars represent mean +/- SD). Widefield microscopy of C. elegans fed (c) GFP expressing Agrobacterium or (d) non fluorescent Agrobacterium.

“Statistics

The distribution of all experimental data was tested using the D'Agostino and Pearson omnibus normality test. Two-tailed non-parametric Mann-Whitney tests were used to assess significance between independent samples from two groups not showing a Gaussian distribution. The Kruskal-Wallis test with Dunn’s post hoc test was used to test significance between three or more independent groups of data not showing a Gaussian distribution. Statistical analysis was carried out using GraphPad Prism Software.**** indicates p≤0.0001, p ** indicates p≤0.01, * indicates p≤0.05. Where no significance is indicated in a figure, p>0.05.”

3. Displaying a control worm in Fig. 3c would help prove that this is a specific fluorescent signal, and not just autofluorescence.

Thank you for your suggestion. I have added a control image to Fig. 3c to show an indication of background autofluorescence in this channel. At this specific exposure there is essentially no autofluorescence in the control worm.

4. The researchers do not mention how often the fluorescence was observed, or under which conditions this was the case (they state "in certain conditions").

Thank you for highlighting this. I have created S1 Table where I provide details of the experimental parameters tested for each T-binary vector. Here I highlight the occasions where atypically bright fluorescence was observed. Representative images for these occasions of bright fluorescence are displayed in S3 Fig.

“

I have also edited the text

“On two separate occasions, worms fed *Agrobacterium* with T-DNA designed to introduce wrmScarlet expression exhibited remarkable fluorescence (Fig 3c). This pattern of atypically bright fluorescence was also observed on 10 occasions with a construct (pmyo3::mCherry::unc-54), where the fluorescent gene was designed to contain introns to preclude any expression of the fluorescent gene by the *Agrobacterium* (Fig. 3c). The conditions used to obtain these results are highlighted in S1 Table. On these twelve occasions , this phenotype was displayed by all *C. elegans* observed, including in the P0 and the F1 generation (S3 Fig), however this bright fluorescence was not consistently reproducible, across different experiments even when these conditions were reproduced.”

S1 Table

5. In Fig. 4 it is stated that n>6, but it is not clear to me what exactly this refers to - number of worms, number of replicates (each replicate containing how many worms?), …

Thank you, I have modified the figure legend to explain that each replicate represents an average of 3 worms per well.

Fig. 4. Locomotion of *C. elegans* fed on *Agrobacterium* control strain, or strains containing T-binary vectors engineered to paralyse *C. elegans*. (a) Frequency of forward motion, (b) frequency of paused motion (Kruskal–Wallis test with Dunn’s post hoc test, n≥6, boxes represent IQR, line shows median value). Each replicate represents an average of 3 worms per well.

6. While Table 1 gives a comprehensive overview of all conditions tested, it isn't clear to me which combinations were tested, which might be useful information for future research. Moreover, no data or statistics are shown, it is just said that "none of the tested conditions or their combinations resulted in successfully transformed C. elegans".

Thank you for this feedback, to address this I have added S1 Table, which provides a detailed overview of all the combinations of conditions tested for each T-binary vector. In order to simplify the presentation, I have replaced the previous Table 1 with a schematic (Fig. 5) that visually outlines the conditions that were varied during the experiments. The full details of all tested conditions are now available in S1 Table, which should help facilitate the replication of these experiments.

Fig 5

7. It might be an issue with uploading the figures, but the resolution in the version I received is much too low.

Thank you, I wasn’t aware of this, I have double checked the resolution and improved where necessary, I will make sure these maintain their resolution for the final submission.

I hope my feedback is useful to the authors, and they will soon be able to publish their results.

Reviewer #3: In this manuscript (PONE-D-24-38923), Warren et al. present data from their attempt to establish a platform for the genetic transformation of C. elegans using Agrobacterium. This bacterial strain is a natural plant pathogen, primarily used for genetic transformation of plants, with limited studies demonstrating its use for transformation in other kingdoms and even human cell lines. The authors argue that conventional methods for genetic transformation of C. elegans (microinjection and biolistic bombardment) lack the potential for high-throughput and timely generation of C. elegans mutant libraries. To address this limitation, the authors propose using different Agrobacterium strains, alongside specific constructs and screening conditions, as a food source for worms to develop a simpler and more efficient high-throughput transgenesis protocol. However, as the authors acknowledge, their attempts were unsuccessful. Nonetheless, they present their data to support the scientific community by providing constructs, conditions, and optimization suggestions, aiming to serve as a foundation for future successful endeavors.
 Also, using established systems for phenotyping C. elegans behavior, the authors tested different constructs with genes responsible for inducing paralysis. Despite these constructs failing to induce paralysis due to unsuccessful transformation from Agrobacterium, the authors assessed worm behavior in terms of forward and paused motion frequencies.
 Overall, the manuscript introduces a novel yet unsuccessful approach for gene transfer from Agrobacterium to C. elegans. Given the significance of C. elegans as a model organism in genetic studies, a contribution acknowledged by four Nobel Prizes, developing a simple and high-throughput method for genetic manipulation is of immense interest. While the authors provide an honest representation of their data, some claims require justification to enhance the reliability of the study and ensure its suitability for publication. Below, I provided some suggestions to improve the manuscript’s rigor and address gaps in study design and presentation.

 Major Points:
 1. The authors state in the Results section: “In certain conditions, worms fed Agrobacterium containing T-DNA designed to introduce wrmScarlet expression did display remarkable fluorescence (Fig. 3c).” Additionally, they claim: “Bright fluorescence appeared to localize to the pharynx, the vulva, and two regions on the ventral side of the nematode approximately 100 μm anterior and posterior to the vulva, consistent with the spermatheca.” Similar claims appear in the Discussion, suggesting that Agrobacterium has the potential to target the germline of C. elegans for generating heritable transformants: “Despite our lack of success, we have observed Agrobacterium triggered fluorescence in the reproductive tissues of the vulva and spermatheca, suggesting that Agrobacterium has the potential to target the germline of C. elegans for the generation of heritable transformants.” and “This indicates that Agrobacterium are interacting at these locations, to trigger fluorescence, rather than accumulating directly. This finding suggests that Agrobacterium-mediated transformation is a promising approach since Agrobacterium are able to access reproductive tissues suggesting a propensity to target the germline of C. elegans for the generation of heritable transformants.”
 Although the authors mentioned that the data presented was not always reproducible, the data presented in Fig. 3c do not convincingly support these claims. The fluorescence attributed to the spermatheca is based on rough distance calculations. Additionally, the control worm in Fig. 3a shows autofluorescence comparable to the foci observed in Fig. 3c.
 • To substantiate this claim, the authors should:

o Provide additional evidence, such as staining with DAPI to detect sperm cell nuclei or using differential interference contrast (DIC) optics to superimpose fluorescent channels on reproductive tissues. These methods would help verify whether the fluorescence observed corresponds specifically to the spermatheca and support the claim of targeted interaction by Agrobacterium.

Thank you for your feedback. In response to your comments, I have made the following revisions:

Fluorescence localization to Spermatheca

I appreciate your suggestion to provide additional evidence for the localization of fluorescence in the reproductive tissues. To address this, I have included S4 Fig, which superimposes the dsRed fluorescent channel with brightfield images to help identify the regions of interest. Unfortunately, we were unable to reproduce the fluorescent phenotype consistently, and as such, we cannot include differential interference contrast (DIC) optics.

I also attempted DAPI staining to identify sperm cell nuclei, as you suggested, but this did not help clarify the localization of the fluorescence. As a result, I have removed all references to fluorescence in the spermatheca and revised the manuscript to describe the fluorescence as occurring in “two regions on the ventral side of the nematode, approximately 100 μm anterior and posterior to the vulva.” DAPI images are shown below, since these did not help identify the localisation of the fluorescence I have chosen not to include these in the paper.

Autofluorescence in Control Worms

Regarding the autofluorescence observed in control worms (Fig. 3a), I acknowledge the similarity between the autofluorescence in these controls and the foci seen in the experimental worms (Fig. 3c). I have now highlighted this observation in S4b Fig, where I compare autofluorescence in control worms fed *Agrobacterium* lacking T-DNA with the fluorescence patterns seen in worms fed *E. coli*. These results suggest that *Agrobacterium* itself might trigger autofluorescence in specific regions, complicating the interpretation of fluorescence as a potential T-DNA expression signal. Given this complexity, we explored other T-DNA constructs (e.g., BFP and T-DNA designed to paralyze worms). Unfortunately, we did not observe successful fluorescence with BFP, nor did we observe paralysis. Based on these findings, we interpret the results as indicative of an unsuccessful transformation. To allow other researchers to fully evaluate our results, I have included images that show striking fluorescence in the presence of Agrobacterium, while also emphasizing the uncertainty regarding the nature of this fluorescence (S4a Fig).

S4 Fig

Here is the modified section of text

“Here we have failed to generate transgenic *C. elegans* using *Agrobacterium*-mediated transgenesis, however in some conditions we have observed intense fluorescence in specific regions of the worms, such as the vulva and two regions anterior and posterior to the vulva (Fig. 3c, S4). While this fluorescence is noticeably brighter, it is located in comparable regions where autofluorescence has been observed in control *Agrobacterium* lacking a T-binary vector (Fig3. a). We have not observed this same pattern of autofluorescence in *C. elegans* fed *E. coli* (S4 b). Using GFP-expressing *Agrobacterium* we demonstrated that the *Agrobacterium* localise within the pharynx and intestine of *C. elegans* with no noticeable accumulation within these regions where we have observed fluorescence (Fig. 2c). This indicates that the observed fluorescence is not caused by *Agrobacterium* accumulation, but may result from an interaction with the bacteria, complicating the interpretation of the red fluorescence as a potential T-DNA expression signal.”

Finally, in order to clarify the difference in autofluorescence observed between media types, I have modified Figure 3 to exclude autofluorescence data and have placed this information in S2 Fig, alongside quantification of autofluorescence observed in worms fed Agrobacterium on two different media types.

Brightfield	DAPI	Merge

o Reevaluate the conclusion that the observed fluorescence is a direct effect of GFP-expressing bacteria. Without stronger evidence, the assertion that Agrobacterium has potential for germline transformation is misleading.

I have changed the text to say

“This indicates that the observed fluorescence is not caused by *Agrobacterium* accumulation, but may result from an interaction with the bacteria”

I have removed any claim that this suggests potential for future germline transformation.

2. The authors state that “C. elegans were able to survive and reproduce on A. tumefaciens, as demonstrated by the presence of progeny after 4 days.” However, no supporting data are provided. The ability to produce viable progeny is crucial for successful genetic transformation.
 • It is recommended that the authors conduct progeny assays to assess the number and viability of offspring when worms are fed on A. tumefaciens compared to E. coli. Include this data as supplemental material to strengthen the study.

Thanks for this suggestion. I have carried out a survival assay to demonstrate the *C. elegans* fed *Agrobacterium* have a comparable survival rate to *C. elegans* fed *E. coli*. Similarly I carried out a progeny assay where I observed that *C. elegans* fed *Agrobacterium* were able to produce viable progeny in a similar time frame to *C. elegans* fed *E.coli* (Eggs of the F1 and F2 generation, are actually layed approximately 1 day earlier on worms fed *agrobacterium* than worms fed *E. coli*). Because of this I also carried out a chemotaxis assay to see if *C. elegans* are preferentially attracted to *Agrobacterium* or *E. coli*, but there was no significant difference between the two. All of this is reported in S5 Fig. I have updated the main body of the text to reflect this.

*“C. elegans* were able to survive and reproduce on *Agrobacterium*, with survival comparable to *C. elegans* on *E. coli* (S5a). *C. elegans* were able to produce viable progeny on *Agrobacterium*, with eggs visible 3 days after refeeding L1s, and F1 progeny hatching 4 days after refeeding (S5b). Eggs from the F1 and F2 generation were laid earlier in *C. elegans* fed *Agrobacterium* (induced or uninduced) compared to *C. elegans* fed *E. coli* (p≤0.01 Kruskal–Wallis test with Dunn’s post hoc test (S5b), however, no attraction to *Agrobacterium* over *E. coli* was observed from a chemotaxis assay (p<0.05, Unpaired Mann-Whitney test) (S5c).”

S5 Fig

3. While the authors’ attempts at gene transfer were unsuccessful, including data on the general transformation efficiency of A. tumefaciens would help assess the feasibility of this approach and provide a benchmark for evaluating its potential in C. elegans. Including this information as supplemental data could help the scientific community better evaluate the feasibility of the proposed approach.

*Agrobacterium* mediated transformation of plant cells is highly efficient. I have referenced a paper (Carlson etal. 2023) where they quantify the efficiency of agrobacterial delivery in Nicotiana benthamiana.

“Given the high efficiency of *Agrobacterium* transformation in plant systems, as demonstrated by the ability to achieve near-simultaneous delivery of over 20 unique T-DNAs to a given plant cell (39), we believe *Agrobacterium* holds significant potential for use in *C. elegans* transformation.”

4. Table 1 lists “Conditions Screened for Agrobacterium Induction and Co-incubation with C. elegans.” While potentially useful, the table lacks important details, likely due to insufficient study design.
 • A more comprehensive table, including key experimental parameters and results, would improve the manuscript’s utility for other researchers.

Thank you for this feedback, to address this I have added S1 Table, which provides a detailed overview of all the combinations of conditions tested for each T-binary vector. In order to simplify the presentation, I have replaced the previous Table 1 with a schematic (Fig. 5) that visually outlines the conditions that were varied during the experiments. The full details of all tested conditions are now available in S1 Table, which should help facilitate the replication of these experiments.

Minor Points:
 1. Reference 14, a master’s thesis analyzing host-pathogen interactions between Agrobacterium and C. elegans, does not include experiments on genetic transformation. However, this reference (although not peer-reviewed), suggests that C. elegans can survive on A. tumefaciens as a sole food source, as shown by longevity assays.
 • The current manuscript evaluates worm crawling speed and length when grown on A. tumefaciens. Since the cited reference includes viability assays, adding comparative data on worms grown on E. coli would provide better context for these observations. Additionally, examining germline morphology, cell migration, or other anatomical or physiological factors could offer deeper insights into the survival and adaptation of C. eleganswhen fed exclusively on A. tumefaciens.

Thank you for this feedback, I agree that it is important to evaluate the viability of worms fed on Agrobacterium. I have conducted a survival assay to demonstrate the *C. elegans* fed *Agrobacterium* have a comparable survival rate to *C. elegans* fed *E. coli*. Similarly I carried out a progeny assay where I observed that *C. elegans* fed *Agrobacterium* were able to produce viable progeny in a similar time frame to *C. elegans* fed *E.coli* (Eggs of the F1 and F2 generation, are actually layed approximately 1 day earlier on worms fed *agrobacterium* than worms fed *E. coli*). Because of this I also carried out a chemotaxis assay to see if *C. elegans* are preferentially attracted to *Agrobacterium* or *E. coli*, but there was no significant difference between the two. All of this is reported in supplementary figure 5. I have updated the main body of the text to reflect this.

*“C. elegans* were able to survive and reproduce on *Agrobacterium*, with survival comparable to *C. elegans* on *E. coli* (S5a). *C. elegans* were able to produce viable progeny on *Agrobacterium*, with eggs visible 3 days after refeeding L1s, and F1 progeny hatching 4 days after refeeding (S5b). Eggs from the F1 and F2 generation were laid earlier in *C. elegans* fed *Agrobacterium* (induced or uninduced) compared to *C. elegans* fed *E. coli* (p≤0.01 Kruskal–Wallis test with Dunn’s post hoc test (S5b), however, no attraction to *Agrobacterium* over *E. coli* was observed from a chemotaxis assay (p<0.05, Unpaired Mann-Whitney test) (S5c).”

S5 Fig

Reviewer #4: This manuscript investigates the potential of using Agrobacterium tumefaciens for high-throughput genetic transformation of C. elegans. The authors demonstrate that worms can survive and reproduce using Agrobacterium as a food source, and sporadic fluorescence in reproductive tissues suggests possible germline targeting. However, routine transgenesis was not achieved. The study explores various transformation protocol parameters to facilitate gene transfer, but these efforts did not yield successful transformation.

 The low throughput of transgenesis remains a significant bottleneck in C. elegans genetics. The authors' attempts, although not yet successful, are valuable to the community. Despite the lack of positive results, this manuscript provides a well-documented account of experimental procedures, optimization strategies, and a useful resource of T-binary constructs, offering insights for future attempts.

 Major Comments：
 1. Line 99: The authors observed progeny after 4 days on A. tumefaciens. In my lab, N2 animals begin laying eggs at 3 days of age on E. coli at 20°C. Are there differences in the growth rates of animals fed on A. tumefaciens compared to E. coli? Measuring growth rates could provide a complementary metric to length and speed, offering a more comprehensive fitness assessment for animals cultured on different food sources.

Thanks for this suggestion. To clarify differences in developmental speed of C. elegans fed on Agrobacterium or E. coli, I have carried out a Progeny assay. I observed that C. elegans fed Agrobacterium are able to produce viable progeny in a similar time frame to C. elegans fed E.coli. Eggs of the F1 and F2 generation, were actually layed 1 day sooner in worms fed agrobacterium than worms fed E.coli. Because of this result I also carried out a chemotaxis assay to see if C. elegans are preferentially attracted to Agrobacterium over E. coli, but there was no significant difference between the two. All of this is reported in supplementary figure 5. I have updated the main body of the text to reflect this.

*“C. elegans* were able to survive and reproduce on *Agrobacterium*, with survival comparable to *C. elegans* on *E. coli* (S5a). *C. elegans* were able to produce viable progeny on *Agrobacterium*, with eggs visible 3 days after refeeding L1s, and F1 progeny hatching 4 days after refeeding (S5b). Eggs from the F1 and F2 generation were laid earlier in *C. elegans* fed *Agrobacterium* (induced or uninduced) compared to *C. elegans* fed *E. coli* (p≤0.01 Kruskal–Wallis test with Dunn’s post hoc test (S5b), however, no attraction to *Agrobacterium* over *E. coli* was observed from a chemotaxis assay (p<0.05, Unpaired Mann-Whitney test) (S5c).”

2. Lines 123–126: The meaning of "in certain conditions" is unclear. Could the authors specify what these conditions entail? Describing them in detail is crucial, as the factors causing Agrobacterium to target specific regions could inform strategies for successful gene delivery.

Thank you for highlighting this. I have created a supplementary Table 1 where I provide details of the experimental parameters tested for each T-binary vector. Here I highlight the occasions where atypically bright fluorescence was observed. Representative images for these occasions of bright fluorescence are displayed in S3.

 “

I have also edited the text

“On two separate occasions, worms fed Agrobacterium with T-DNA designed to introduce wrmScarlet expression exhibited remarkable fluorescence (Fig 3c). This pattern of atypically bright fluorescence was also observed on 10 occasions with a construct (pmyo3::mCherry::unc-54), where the fluorescent gene was designed to contain introns to preclude any expression of the fluorescent gene by the Agrobacterium (Fig. 3c). The conditions used to obtain these results are highlighted in S1 Table. On these twelve occasions when this fluorescence intensity was observed, this phenotype was displayed by all C. elegans observed, including in the P0 and the F1 generation (S3 Fig), however this bright fluorescence was not consistently reproducible, across different experiments even when these conditions were reproduced.”

3. Line 162: The sample size for behavioral phenotyping is too small to draw solid conclusions. I recommend increasing the sample size, ideally doubling it, and reanalyzing the data to strengthen the conclusions.

I appreciate the reviewer’s concern regarding the sample size for behavioral phenotyping. In this experiment, we conducted six independent replicates, with each replicate representing an average of three worms per well. We believe this sample size is appropriate for detecting significant differences in locomotion behavior. However, I acknowledge that our initial description may not have been sufficiently clear. To address this, I have revised the figure legend to explicitly clarify what each data point represents.

"Fig. 4. Locomotion of C. elegans fed on Agrobacterium control strain or strains containing T-binary vectors engineered to paralyze C. elegans. (a) Frequency of forward motion, (b) frequency of paused motion. (Kruskal–Wallis test with Dunn’s post hoc test, n≥6, boxes represent interquartile range [IQR], line shows median value). Each independent replicate represents an average of three worms per well."

4. Lines 225-227: The authors suggest introducing male worms to enhance Agrobacterium's entry through the vulva. Running a quick experiment to test this hypothesis would provide valuable supporting evidence.

Thanks for the suggestion, I agree that this is worth testing and have conducted the proposed experiment by introducing male worms to assess whether this enhances Agrobacterium entry through the vulva. I used the strain CB4088 (him-5(e1490) V.) to provide a high incidence of males. I carried out these experiments using either GFP expressing Agrobacterium (to monitor localisation of the Agrobacterium within the worm), and with Agrobacterium containing T-binary vectors for the introduction of mCherry or wrmScarlet into the worms. Our results showed no difference compared to N2 worms.
Images shown below (not included in the paper)

I have updated the text in the paper to demonstrate that we have attempted this approach

“Given that the vulva may serve as an entry point for Agrobacterium, we also explored Agrobacterium-mediated transgenesis in *C. elegans* using the CB4088 (*him-5(e1490) V*.) strain, which exhibits a high incidence of males. Our aim was to facilitate mating between male worms and hermaphrodites, thereby enhancing agrobacterial entry through this route and potentially increasing access to the germline. However, none of the tested conditions or their combinations resulted in successfully transformed C. elegans in our hands.”

5. General Thought for Future Research: Before testing on whole animals, it might be worthwhile to perform ex vivo infection tests using cultured C. elegans germ cells. This approach could help refine conditions for optimal Agrobacterium attachment and gene delivery into cells, providing a more controlled environment for initial explorations.

Thank you for this suggestion, I hope that labs with expertise in working with cultured *C. elegans* germ cells will be interested in testing this in the future. I have modified the text to highlight that the constructs we are making available could also be used for optimising in cultured germ cells.
“The constructs designed here (Table 1) are suitable for *in vivo* genetic transformation of C. elegans animals, or transformation of *ex vivo* cultured *C. elegans* germ cells and should provide a useful resource for optimizing *Agrobacterium*-mediated transformation of *C. elegans.“*

Minor Comments:
 1. Lines 112–113: These lines should not be divided by a new line, as they are part of the same sentence.
 2. Line 143: Replace "pmyc3" with "pmyo3." done 3. Line 207: A full stop is missing before "Here we"
 4. Line 280: Replace "form" with "from."

Thank you for identifying these typos, these have been corrected in the manuscript
